# Spatial analysis and visualization of global data on multi-resolution hexagonal grids

**DOI:** 10.1007/s42081-020-00077-w

**Published:** 2020-04-25

**Authors:** T. Stough, N. Cressie, E. L. Kang, A. M. Michalak, K. Sahr

**Affiliations:** 1 NASA Jet Propulsion Laboratory, California Institute of Technology, Pasadena, CA USA; 2 National Institute for Applied Statistics Research Australia, University of Wollongong, Wollongong, Australia; 3 Division of Statistics and Data Science Department of Mathematical Sciences, University of Cincinnati, Cincinnati, OH USA; 4 Department of Global Ecology, Carnegie Institution for Science, Stanford, CA USA; 5 Department of Computer Science, Southern Oregon University, Ashland, OR USA

**Keywords:** Geographic Information Science, Discrete global grids, Raster data modelling, Spatial analysis, Remote sensing

## Abstract

In this article, computation for the purpose of spatial visualization is presented in the context of understanding the variability in global environmental processes. Here, we generate synthetic but realistic global data sets and input them into computational algorithms that have a visualization capability; we call this a simulation–visualization system. Visualization is key here, because the algorithms which we are evaluating must respect the spatial structure of the input. We modify, augment, and integrate four existing component technologies: statistical conditional simulation, Discrete Global Grids (DGGs), Array Set Addressing, and a visualization platform for displaying our results on a globe. The internal representation of the data to be visualized is built around the need for efficient storage and computation as well as the need to move up and downresolutions in a mutually consistent way. In effect, we have constructed a Geographic Information System that is based on a DGG and has desirable data storage, computation, and visualization capabilities. We provide an example of how our simulation–visualization system may be used, by evaluating a computational algorithm called Spatial Statistical Data Fusion that was developed for use on big, remote-sensing data sets.

## Introduction

As satellite technologies for Earth observation have advanced over the past decades, the volume and complexity of geophysical data collected by space-based instruments has grown, and so have the challenges of interrogating these data and drawing quantitative conclusions from them. Large-scale computational algorithms that transform data through many stages, from raw bits to meaningful information, are required to realize an order-of-magnitude increase in scientific return. However, those algorithms necessarily incorporate modeling assumptions and computational approximations that may lead to artifacts that may in turn compromise scientific conclusions. Visualization plays a key role in understanding and quantifying geophysical artifacts. The geolocational aspects of remote-sensing data make them natural to visualize and interactively explore through maps.

The usual mechanism for evaluating computational algorithms is a simulation experiment (SE): simulated data with known properties are used to generate synthetic input to the algorithm of interest, and the algorithm’s outputs are compared to the corresponding “true” values obtained from the simulated data. Implementing SEs for algorithms that are designed to run on massive satellite data sets can be challenging for at least two reasons. First, many geophysical processes of interest vary continuously in space, requiring very-high-resolution simulations to realistically mimic them. Moreover, realism also requires that scientific knowledge of the underlying geophysics be brought to bear by enforcing some form of consistency between the simulated data and how we expect the true system to behave. This goal can be achieved, for example, by ensuring that the simulated data be consistent with the output of a coarse-resolution, geophysical process model. This means that the simulations must be consistent across scales, not only with respect to mean structure but also with respect to spatial covariance. Second, observed data collected by satellite remote-sensing instruments represent incomplete aggregates over different spatial supports, with measurement errors superimposed. The SE must recreate this by averaging the synthetic field over an instrument’s ground footprints, or sampling from the field if the footprints are smaller than the resolution of the simulated field. These operations must be performed in a way that recreates the spatial sampling and error characteristics of real satellite instruments.

Both these problems require that the simulated field exhibits reasonable spatial coherence and variability. One way to achieve this is through a fine-resolution spatial statistical model that respects the output of a coarse-resolution physical (deterministic) model. By this, we mean that the parameters of the statistical model are set in such a way that when the simulated field is aggregated up to the coarse resolution of the physical model, it is guaranteed to reproduce the output of the physical model. We call this constrained-parameter-fitting procedure *calibration*, and we use *conditional simulation* (e.g., Cressie 1993, Ch. 3) to simulate from the calibrated model. Here, the computational algorithm which we use to illustrate our approach is Spatial Statistical Data Fusion (SSDF; Nguyen et al. 2012), which ingests two or more massive, heterogeneous, remote-sensing data sets and produces an optimal estimates of the underlying field. Note that this is different from the following validation exercise, where the computational algorithm’s spatial output is compared to actual observations that are sparsely distributed in space at the resolution of the output. Crowell et al. (2019) have produced an attractive visualization tool for global flux inversion of carbon dioxide, for this type of problem.

The visualization challenge is to display the massive, fine-resolution conditional simulation and the equally massive output of SSDF, so that they can be compared. Both data sets are global and are expected to reproduce large-scale and small-scale spatial structures. The visualization system must be able to render these features without geometric distortion, and it must be capable of zooming in and out, so that features and possible artifacts can be explored at a variety of scales.

A number of systems and software tools for multi-resolution geographic visualization already exist. Google Earth displays and allows for pyramid-based multi-resolution zoom. However, it uses a cylindrical projection that causes distortions in both appearance and, crucially for us, in its representation of spatial relationships. The cylindrical projection creates a non-uniform tiling of Earth’s surface, with tiles becoming smaller near the poles. This distorts the spatial–statistical properties of fields whose units are “per unit area.” Ladstdter et al. (2010) has developed a system for exploring large climate data sets using interactive visualization and simple statistical tools. This system uses a cylindrical projection and does not perform computations on the sphere. Other tools designed for global data sets (e.g., The Global Climate Change Viewer Alder et al. 2013; Climate Wikience Rodriges Zalipynis et al. 2011) typically display data at resolutions that are too coarse for our purposes and use latitude–longitude grids whose tiles are again of unequal area. While they often possess simple computational tools, they do not typically include downscaling to the finer resolutions, where our interest lies.

The HEALPix (Hierarchical Equal Area isoLatitude Pixelization) (Górski et al. 2007) system represents data at multiple resolutions, with storage and computation on the sphere. However, it does not provide a visualization capability by itself, and it does not use hexagonal tessellations of the sphere, which are ideal for spatial statistical inference (Olea 1984). This article describes our approach to visualizing global data on multi-resolution grids.

Our simulation–visualization system is in effect a Geographic Information System (GIS) that combines four key technologies: (1) a multi-resolution, statistical process model, calibrated to the output from a coarse-resolution deterministic model; (2) the Discrete Global Grids (DGG) software package for tessellating the globe with a hierarchy of nested hexagonal grids to provide a system of multi-resolution supports for prediction; (3) an enhanced indexing system for cells of spherical hexagonal grids and for mapping the cells onto a flat plane, so that the spatial–statistical process model can be used without geometric distortion; and (4) a visualization platform for multi-resolution, interactive visualization of the simulated field and the computational algorithm being evaluated. In Sect. 2, we describe these four technologies and how we adapted and integrated them for our purposes. Section 3 is a case study showing how we used our system to visualize (a) simulated fine-resolution fields produced by conditional simulation, (b) synthetic instrument observations constructed from the simulated field, and c) the output from SSDF. Finally, in Sect. 4, we offer some conclusions about the efficacy of our system and a discussion of future work.

## Algorithms and methods

We have combined four component technologies to create a simulation–visualization system for massive geophysical data sets. In this section, we describe these components and how we have adapted them for our purposes. In Sect. 2.1, we briefly introduce conditional simulation. In our context, it uses a dimension-reducing, multi-resolution spatial statistical model that enables optimal spatial prediction at a variety of spatial resolutions. Those predictions are identified with the hexagonal cells of the DGG, which have certain desirable properties (e.g., equal area) and are described in Sect. 2.2. To exploit DGG’s downscaling and image-processing features, two things are required: a method for flattening spherical grids onto two-dimensional planes, and an efficient indexing system for the grid cells. In Sect. 2.3, we describe the computational algorithms used to satisfy these two requirements. Regarding the visualization platform, our choice was Google Earth, which is a ubiquitous and intuitive interactive visualization environment for multi-scale georeferenced data sets. In Sect. 2.4.2, we describe how we leverage this platform for the exploration of spatial predictions at multiple scales.

### Conditional simulation

Atmospheric processes are defined at every location on the sphere, which is our mathematical abstraction of Earth’s surface. In practice, the surface of the sphere is discretized into a fine-resolution regular grid; we call a generic grid cell a Basic Areal Unit or BAU. Here, we let the BAUs be the hexagons of the DGG at the finest resolution of interest (see Sect. 2.2) and identify each BAU by the latitude and longitude of its center. Let $\varvec{s}$ denote the two-dimensional latitude–longitude center of a BAU. Then a generic spatial–statistical model for the geophysical variable of interest, *Y*, at $\varvec{s}$ is:

1 $$\begin{aligned} Y(\varvec{s})&= \mu (\varvec{s}) + \nu (\varvec{s}) + \xi (\varvec{s}), \end{aligned}$$

where $\varvec{s}$ ranges over the sphere, $\mu (\varvec{s})$ is the large-scale trend, $\nu (\varvec{s})$ is smooth small-scale variation, and $\xi (\varvec{s})$ represents the remaining micro-scale variation. The components on the right-hand side of (1) are assumed to be statistically independent.

Suppose that the total number of BAUs over Earth’s surface is *N*; then, we can form *N*-dimensional vectors for each of the terms in Eq. (1) by simply stacking the terms corresponding to the *N* locations into column vectors. Thus, we can write the entire generic model (1) compactly as:

2 $$\begin{aligned} \mathbf {Y}&= \varvec{\mu } + \varvec{\nu } + \varvec{\xi }. \end{aligned}$$

Cressie and Johannesson (2006, 2008) developed a flexible, nonstationary spatial–statistical model they called the Spatial Random Effects model (SRE; see also Shi and Cressie 2007), and we use that model here for $\varvec{\nu }$ and $\varvec{\xi }$. Specifically, we assume that $\varvec{\mu }$ describes the mean of $\mathbf {Y}$ and that $\varvec{\nu }$ and $\varvec{\xi }$ are independent, zero-mean, multivariate Gaussian distributions, where $\varvec{\nu } = \mathbf {S} \varvec{\eta }$, $\varvec{\eta }$ is a low-dimensional vector of random effects, $\mathbf {S}$ is a known matrix of basis functions, and $\mathrm {var}(\varvec{\xi })$ is diagonal.

To simulate the entire field $\mathbf {Y}$, we use *y* values defined on a coarse-scale grid that represent our scientific understanding of the geophysical processes of interest. These might be output from a finite-element approximation to a physical model. For instance, in Sect. 3, we use the output of the Parameterized Chemistry and Transport Model (PCTM) for $\hbox {CO}_{2}$ concentrations at the resolution of 1$^\circ\, \times$ 1.25$^\circ$ as our coarse-scale *y*-values; these “inform” the simulation on BAUs defined by the finer-resolution DGG resolution-8 hexagons (30 km in diameter). They also are used to estimate the parameters in $\varvec{\mu }$, $\mathrm {var}(\varvec{\mu })$, and $\mathrm {var}(\varvec{\xi })$.

Let the number of coarse-scale grid cells be *M*, and let ${\tilde{\mathbf{Y }}}$ be the associated *M*-dimensional vector of *y*-values. We consider the coarse-scale process to be an integrated version of the underlying geophysical processes, namely:

$$\begin{aligned} {\tilde{\mathbf{Y }}}=\mathbf {A} {} \mathbf {Y} , \end{aligned}$$

where $\mathbf {A}$ is the $M\times N$ incidence matrix that describes the relationship between the BAUs and the coarse-scale grid. The matrix $\mathbf{A}$ is determined by the assignment of each BAU to a unique coarse-scale grid cell.

Models for $\varvec{\mu }$, $\varvec{\nu }$, and $\varvec{\xi }$ result in models for $\tilde{\varvec{\mu }} \equiv \mathbf {A} \varvec{\mu }$, $\tilde{\varvec{\nu }} \equiv \mathbf {A} \varvec{\nu }$, and $\tilde{\varvec{\xi }} \equiv \mathbf {A} \varvec{\xi }$. Consequently, we can “calibrate” choices for $\varvec{\mu }$, $\varvec{\nu }$, and $\varvec{\xi }$ based on the empirical mean and empirical covariance of $\mathbf {Y}$.

Naturally, we would like the simulated values at BAUs to be “consistent” with the physical-model output. At the very least, we require that, when the simulated field from the BAU scale is aggregated up to the coarse scale of the geophysical model, the simulated field reproduces the model output. To achieve this, instead of simulating $\mathbf {Y}$ from its joint distribution obtained from (2), we simulate from the conditional distribution of $\mathbf {Y}$, conditional on the physical-model output. That is, we generate an *N*-dimensional vector $\mathbf {Y}$ from the conditional distribution $\mathbf{Y}$ given $\mathbf{A}{} \mathbf{Y}=\tilde{\mathbf{Y}}$. In obvious notation:

3 $$\begin{aligned}&\mathbf{Y} \vert \mathbf{A} {} \mathbf{Y}= \tilde{\mathbf{Y }} \nonumber \\&\sim \mathrm {Gau}( \varvec{\mu } + \varvec{\Sigma }{} \mathbf{A} ^{\prime }\left( \mathbf{A} \varvec{\Sigma }{} \mathbf{A} ^{\prime }\right) ^{-1}(\tilde{\mathbf{Y }} - \mathbf{A} {\varvec{\mu }}),\varvec{\Sigma } - \varvec{\Sigma }{} \mathbf{A} ^{\prime }\left( \mathbf{A} \varvec{\Sigma }{} \mathbf{A} ^{\prime }\right) ^{-1}{} \mathbf{A} \varvec{\Sigma }), \end{aligned}$$

where $\varvec{\Sigma }\equiv \mathrm {var}(\mathbf {Y} )$, and note that the parameters in (3) are estimated form the data on the coarse-scale grid. This allows us to simulate finer-resolution *y* values consistent with the coarse-resolution output. Note that the conditional simulation defined by (3) requires computation of $(\mathbf {A} \varvec{\Sigma }{} \mathbf {A} ^\prime )^{-1}$, the inverse of an $M\times M$ matrix. We take advantage of the variance–covariance structure resulting from the Spatial Random Effects model and use the Sherman–Morrision–Woodbury formula (e.g., Cressie and Johannesson 2006, 2008) to obtain the inverted matrix, $(\mathbf {A} \varvec{\Sigma }{} \mathbf {A} ^\prime )^{-1}$ with computational complexity of only *O*(*M*).

### Discrete global grids

Discrete Global Grids (DGGs; Sahr et al. 2003) provide an approach to uniformly tiling the sphere with equal-area hexagonal cells at multiple resolutions. Regular polygonal cells are defined on the faces of a regular polyhedron, and these cells are then projected to the sphere using an appropriately designed inverse equal-area projection. Since a base polyhedron has the same topology as the sphere, the topological singularities associated with whole-Earth cylindrical projections are avoided.

**Fig. 1:**
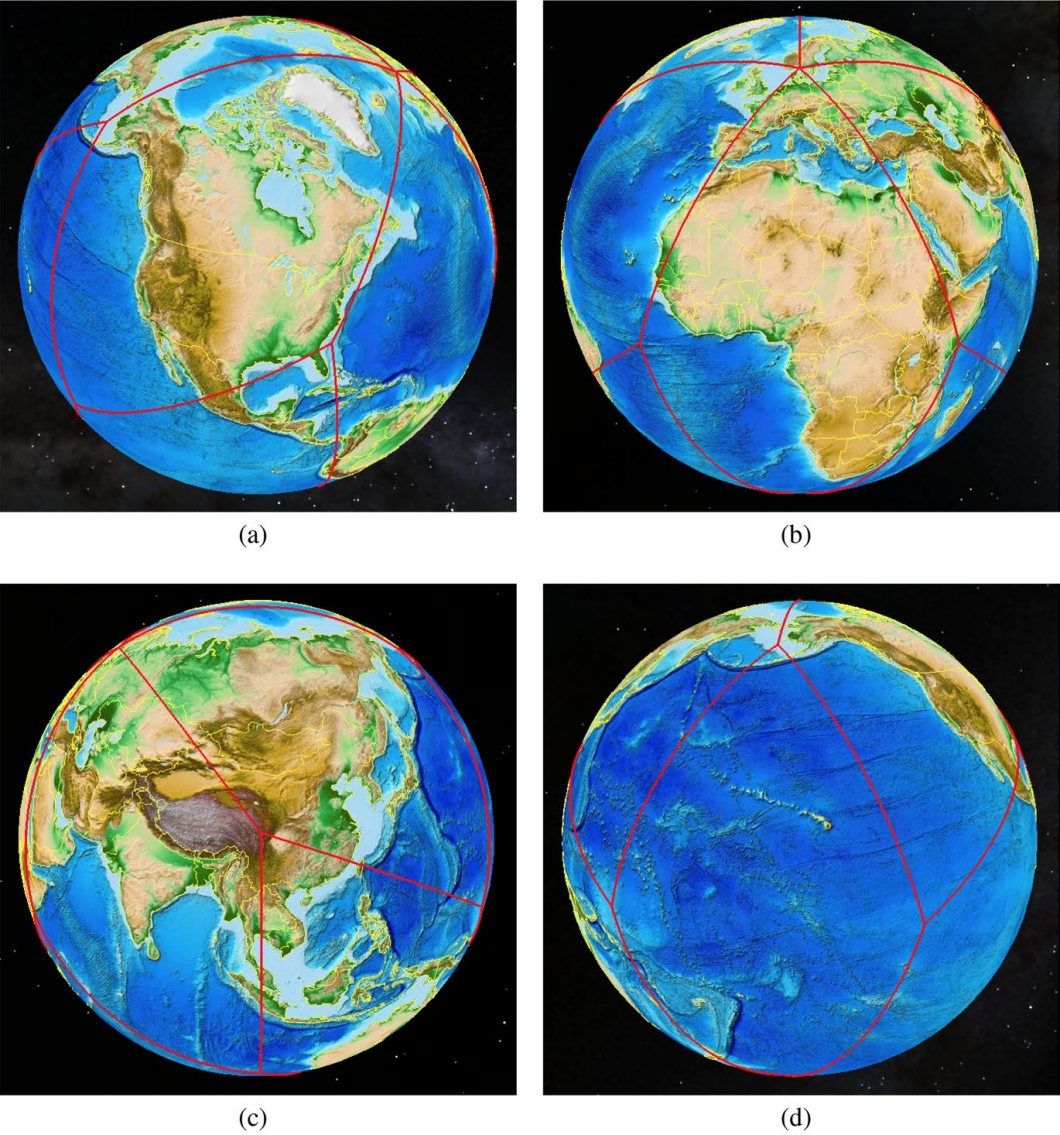
The ISEA Icosahedron wrapped onto the globe. In this figure, quadrants are featured; a quadrant is a diamond made up of two triangular faces

The ISEA4H (Icosahedral Snyder Equal Area aperture 4 Hexagonal) DGG was chosen for this study (Sahr et al. 2003). This DGG is constructed by tiling an icosahedron with cells that are primarily regular hexagons. The icosahedral quadrants are shown from four views around the globe in Fig. 1.

Hexagonal grid cells have numerous advantages over the traditional square grid cells. Hexagons are the most compact regular polygons that tile the plane, and hexagonal cells exhibit unambiguous uniform adjacency. Rasters of hexagonal pixels are 13.4% more efficient at sampling circularly band-limited signals (Petersen and Middleton 1962). For kriging (e.g., Cressie 1993), hexagons have lowest average standard error, lowest maximum standard error, and maximum screen effect (Olea 1984). The article (Sahr 2011) provides a survey of additional advantages of hexagonal grids. It should be noted that it is impossible to tile a polyhedron completely with hexagons; in the case of the icosahedron, the 12 cells centered on the vertices of the icosahedron are pentagons with exactly 5/6 the area of the hexagonal cells.

**Fig. 2:**
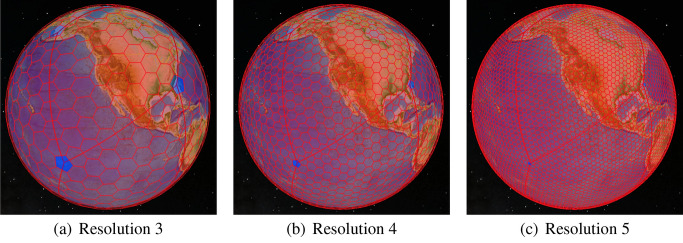
The ISEA4H DGG across multiple resolutions. Notice that the 12 blue cells at the intersections of the icosahedral edges are pentagonal and at the same locations across resolutions

In the ISEA4H DGG, multiple grid resolutions are constructed by introducing, at each resolution, cells that are 1/4 the size of the cells at the next coarsest resolution. The icosahedral version of the Snyder equal area polyhedral projection (Snyder 1992) is used to inversely project the cells from the icosahedral faces to the sphere, preserving equal area at the cost of distorting the shapes of the hexagonal cells. The DGG software provides us with grids at increasingly fine levels of resolution, ranging from 12 7674-km cells at the root of this hierarchy; to 40,962 120-km cells at resolution 6; to 655,362 30-km cells at resolution 8 (the resolution of our BAUs); and to more than 671 million 1-km cells at resolution 13. For example, resolutions 3, 4, and 5 are shown in Fig. 2.

There are two primary ways in which cells on a DGG are indexed. Cells are either given a unique ID, or they are referred to by their icosahedral quadrant (the diamond made up of two triangular faces), numbered 1–10, and a two-dimensional coordinate on that quadrant, q2di (see Fig. 4; quadrants 0 and 11 contain the north and south icosahedral poles, respectively). Both the unique ID and the q2di indexing methods allow any cell on the globe to be referenced, but neither method gives guaranteed information about a cell’s neighbors nor points to an elegant way to store the data in memory while maintaining locality of reference. In the next section, we shall describe a method for storing and indexing the grid that maintains these properties.

### Efficient storage and multi-resolution image processing on global grids

The DGG (Discrete Global Grid) provides a multi-resolution global grid that covers a sphere with equal-area hexagons, modulo 12 pentagons. However, indexing these grid cells in a way that allows efficient storage, computation, and locality of reference is not simple. In this section, we consider the unfolding of the icosahedron, flattening the grid onto the plane, indexing and storing the grid in computer memory, padding the planar representation of the grid to allow for efficient computation, and, finally, pyramiding and multi-resolution issues.

#### Unfolding and flattening

**Fig. 3:**
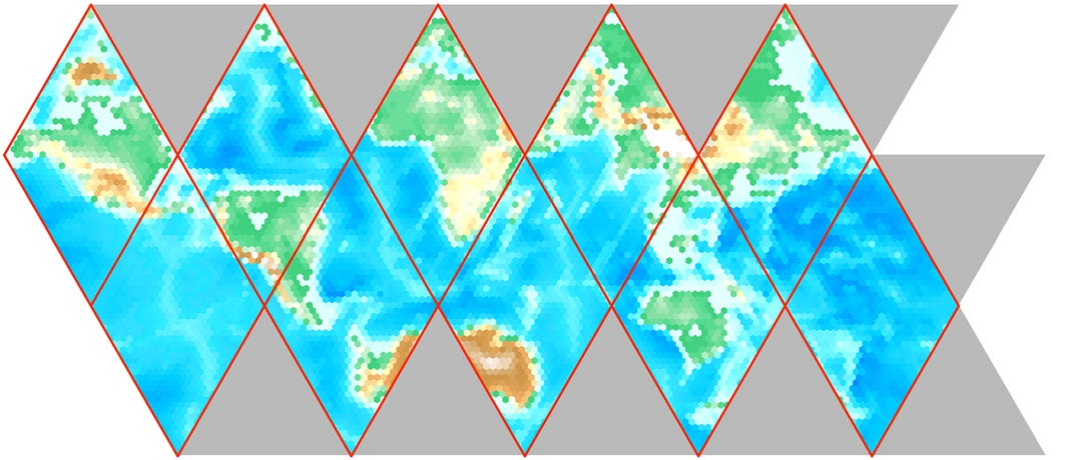
A flattened ISEA4H icosahedron, with a coarse-scaled global topographic map superimposed

**Fig. 4:**
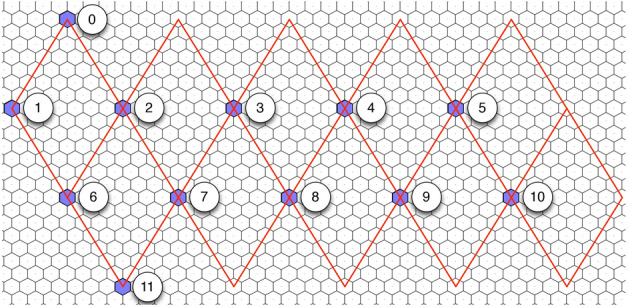
The flattened icosahedron as it maps onto hexagonal graph paper. The intersection points of the icosahedral quadrants are shown inside the blue hexagons next to the numbered circles (colour figure online)

After unfolding the icosahedron shown in Fig. 1, the next step is to flatten the global grid onto a two-dimensional plane that can be easily manipulated and stored; see Fig. 3 and Carr et al. (1997). A key goal in flattening is to achieve an arrangement of grid cells in computer memory that maintains the locality of reference. After removing the topographic content, Fig. 4 shows the underlying hexagons and how the icosahedron is projected onto a sheet of hexagonal graph paper.

It is then necessary to choose an indexing scheme that allows efficient storage and addressing in the flattened grid as well as a method for dealing with the undefined regions, or gores, shown as large gray triangles in Fig. 3.

#### Indexing

**Fig. 5:**
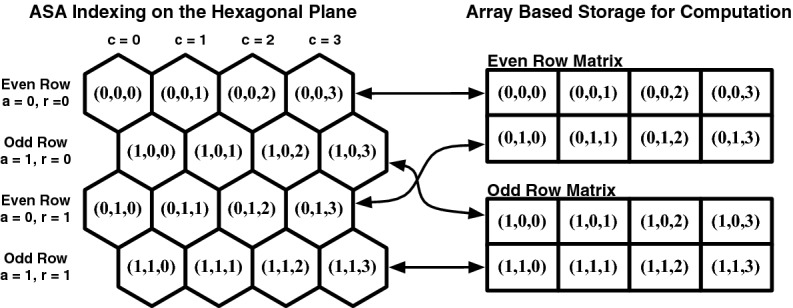
Hexagonal grid separated into two arrays and addressed using Array Set Addressing (ASA)

Array Set Addressing (ASA; Rummelt and Wilson 2011) provides a simple coordinate system with an efficient storage template for planar hexagonal grids. ASA hexagonal grids are divided into two arrays, one for the even rows and one for the odd rows (see Fig. 5).

**Fig. 6:**
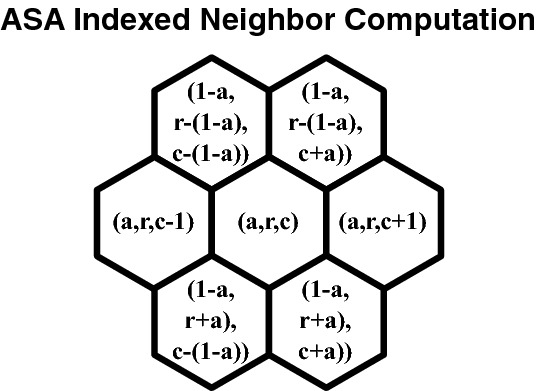
Formulas for Array Set Addressing (ASA) neighbors

The ASA coordinate for any hexagonal cell is indexed by the triple (*a*, *r*, *c*), where ${a \in \{0,1\}}$ specifies which of the two arrays, and the two elements in (*r*, *c*) specify the row and column number, respectively. Critically, using ASA indexing in our system is what allows the quick computation of neighbors that would otherwise be impossible in a standard DGG (see Fig. 6).

The ASA coordinate system also allows fast computation of distances, vectors, and routing on a hexagonal grid. Convolution can be performed using optimized matrix operations on the arrays in memory, allowing fast downsampling, filtering, sampling, and other image-processing operations.

#### Padding and NaN poisoning

**Fig. 7:**
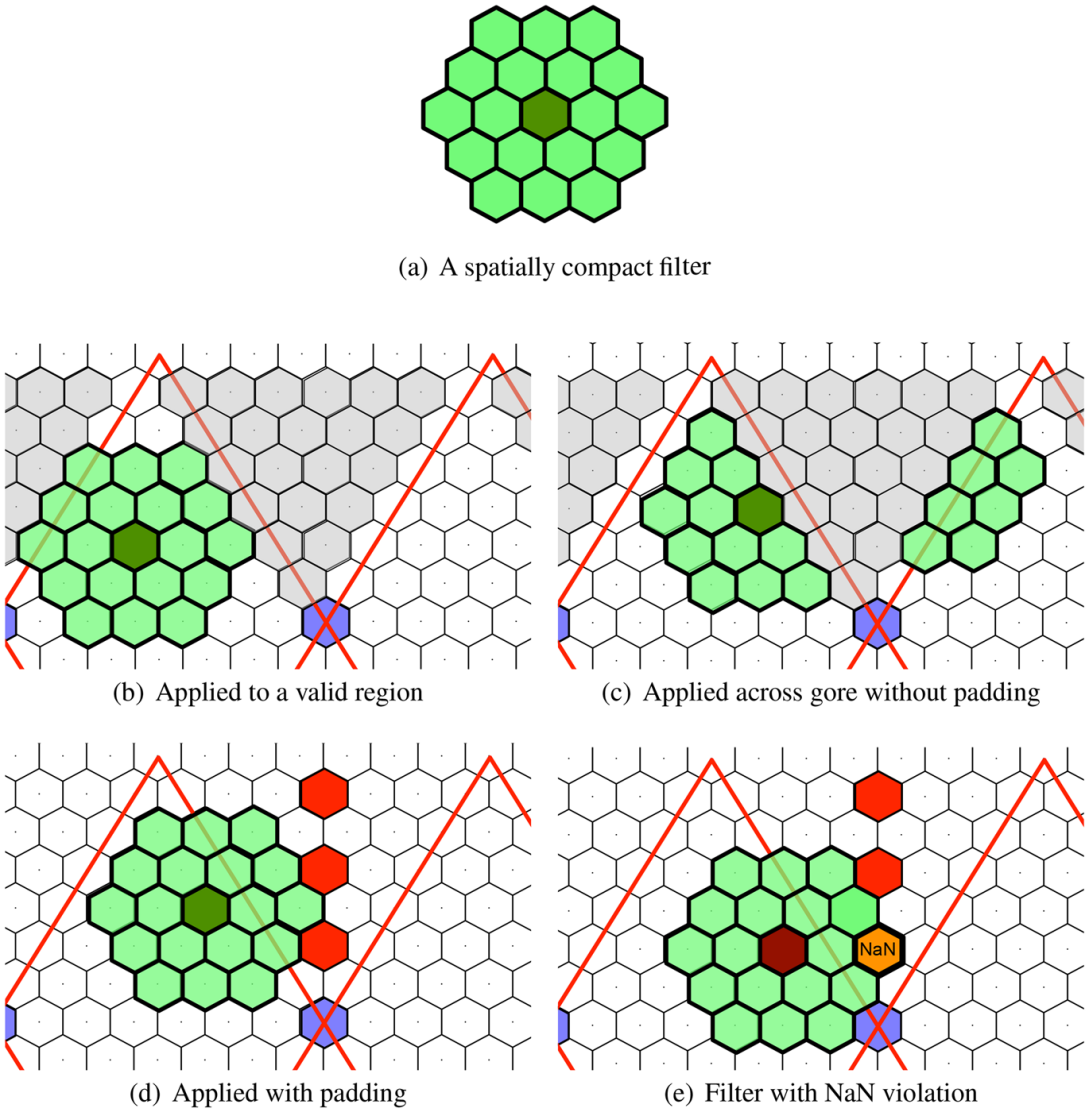
A demonstration showing the application of a matched filter within a region of valid data, across a padded region, and into “NaN-poisoned” cells

The flattened DGG does not completely fill a plane with hexagonal cells. There are gores, or empty locations, in the planar representation of the globe, as well as padding at the edges of the planar image (see Fig. 3). To compute efficiently on this plane, with the topology of the sphere, we pad the gores and edges with the values that would be neighbors to those cells on the folded icosahedron. Unfortunately, it is impossible to pad the entire gore in a consistent way as the mapping breaks down across the centerline of the gore at each pentagonal cell. To detect and deal with computations that involve undefined cells, we pad the centerline of each gore with “NaN” (or Not a Number). The result of this strategy assures that, when computing on the flattened plane, the NaN result will “poison” any computations that include the centerline of a gore. To understand how padding relates to computation on the sphere, we show an example of a spatially compact filter (see Fig. 7a) that might be applied to the global data set.

**Fig. 8:**
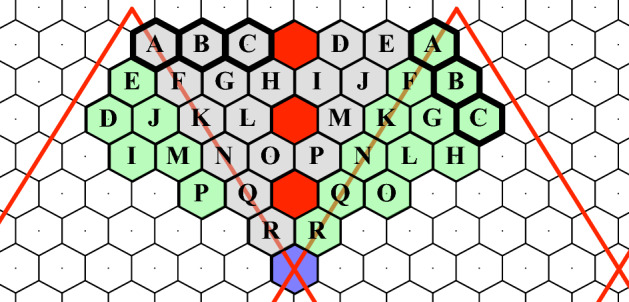
The blue cell and the three red cells along the centerline of the gore are NaN-poisoned. The gray cells in the gore are filled with the values of the valid green cells that they overlap with after folding according to the lettered scheme. Notice how A, B, and C map across to the other side of the fold (colour figure online)

If this filter is applied in the valid region of an unfolded DGG (see Fig. 7b), it is easy to find cells that are contained in the filter. As the filter approaches the gore, its neighbors pass to the other side in a way that requires a special-case computation (see Fig. 7c). However, with padding, the filter can be processed without a special case (see Fig. 7d). As the filter moves closer to the edge of the gore, it encounters a NaN-poisoned cell (Fig. 7e) and produces a NaN result at the center of the filter. In general, there are fewer NaN results with padding than without padding. More details on NaN poisoning and the padding of a representative gore are shown in Fig. 8.

**Fig. 9:**
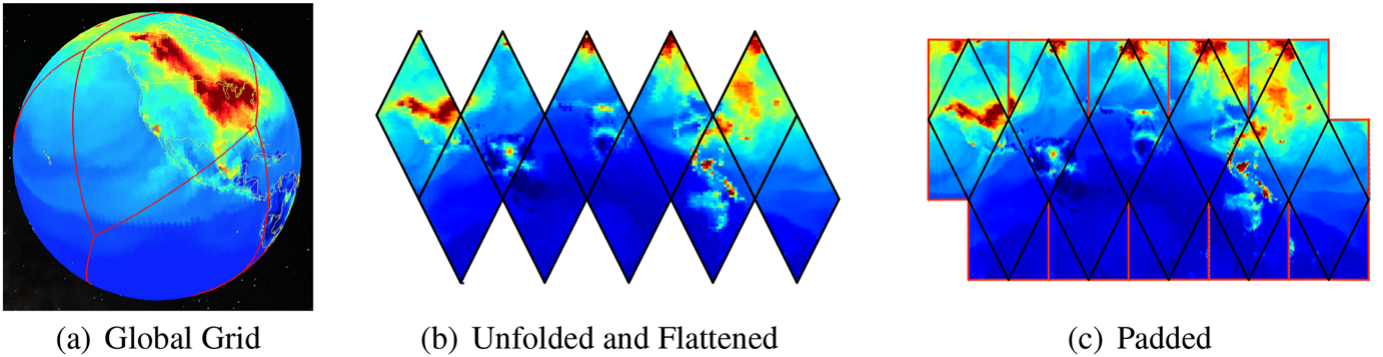
An example of unfolding, flattening, and padding a DGG

In Fig. 9, we show the sequence of unfolding, flattening, and padding a globally gridded data set of $\hbox {CO}_{2}$ in ppm. The DGG is first mapped onto the plane by unfolding the ISEA icosahedron.

Then, cells in the triangular gores are filled in by computing their positions in the folded icosahedron. The red lines show NaN-poisoned cells. Any computation that encounters a NaN-poisoned cell returns a value of NaN and can be computed differently and more slowly by performing convolution a temporary hexagonal array centered on the filter rather than using FFT on the larger array.

This allows us to compute quickly with locality of reference on the vast majority of the sphere via the efficient storage and indexing method described above. The 12 regions on the sphere where special-case processing is required can often be ignored and computed only when needed. Hence, unfolding, flattening, ASA indexing, and NaN poisoning provides a way to move data located on a DGG into arrays in memory that can be operated on efficiently using standard image-processing techniques, with only small modifications.

#### Multi-resolution DGGs, pyramids, and downsampling

**Fig. 10:**
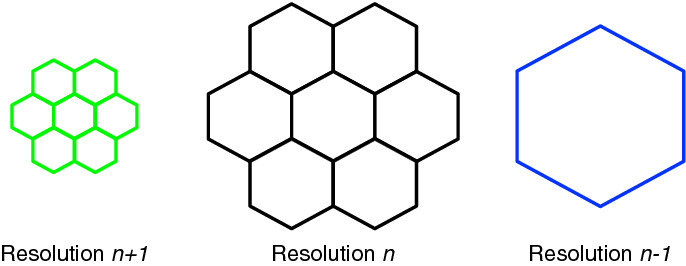
Relationship between levels in a multi-resolution DGG

As mentioned above, DGGs are inherently multi-resolutional with a defined relationship between levels. In the case of the ISEA4H grid that we are using, each cell at a given resolution maps to four cells at the next-finer resolution (see Fig. 2). Due to the nature of hexagonal tiling, cells at the next-finer resolution are not fully nested. Each cell covers one finer cell and half of that cell’s six neighbors, as shown in Fig. 10.

**Fig. 11:**
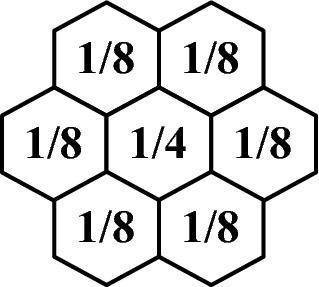
Downsampling kernel that defines data at the next coarsest resolution

In order to build a multi-resolutional pyramid, we begin with the data in resolution *n* and apply the downsampling kernel shown in Fig. 11.

This kernel is applied on the global grid in the ASA addressing space (unfolded, flattened, padded, and NaN-poisoned) using fast FFT-based convolution. Once processed, the resulting ASA array is decimated to leave only values in cells at resolution-$(n-1)$. Next, the resolution $(n-1)$ array is processed to recompute any NaN-poisoned values near the 12 pentagons. This process is repeated until the desired resolution of the data is reached.

The hexagonal structure of the DGG for tessellating the surface of the sphere has many attractive properties, as we have presented in this chapter. While the lack of complete nesting described above is one that is less than desirable, we deal with it through proportional disaggregation (Fig. 11), which in survey-sampling terminology, it is referred to as “raking.” It is an algorithmic approach to a change-of-support problem that has a (Bayesian) statistical justification; see Wikle and Berliner (2005).

### Integrating component technologies

**Fig. 12:**
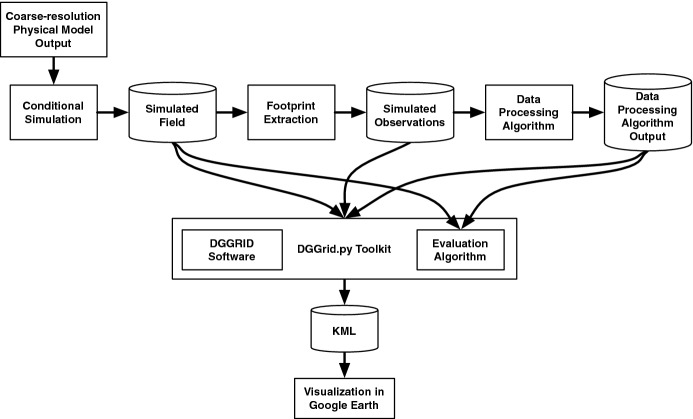
The simulation–visualization system diagram. The Evaluation algorithm calculates and displays a fidelity metric for each hexagonal cell at the resolution of the visualization

Our end-to-end simulation–visualization system is implemented with a python toolkit called DDGrid.py. This toolkit wraps the DGGRID software (Sahr 2011) and implements the data structures and algorithms required to store, manipulate, and visualize simulated fields. This allows us to extract (synthetic) instrument observations, and later to visualize the output of the computational algorithm being evaluated. DDGrid.py leverages the existing optimized image-processing tools from Numpy and SciPy (Peterson 2007) for building multi-resolutional pyramids. It is also used for computing simulated observations by averaging the BAU-level hexagons that coincide with the ground footprints of remote-sensing instruments. Figure 12 is a data-flow diagram showing the main components of this system.

#### Object-oriented Python and C Toolkit

The DGGrid.py toolkit follows the principle of object-oriented design. The DGG is instantiated in objects that represent the entire grid as well as individual hexagons. This object-oriented structure allows us to support many features, like plug-in models for instrument footprints, different data types, and different visualization styles and evaluation functions. The toolkit integrates the global grid structure produced by DGGRID with the unfolded, flattened, padded, NaN-poisoned, and ASA addressable representations. The objects in DGGrid.py map to the topology and cells of the DGG, and each object is capable of producing KML (Keyhole Markup Language) to visualize itself. This allows us to subset the grid into any grouping that we like. We can also use the boundaries of any grid cell to create finer-resolution cells that make up the original grid cell. Together, these features allow the production of easy-to-visualize, multi-resolutional grids.

In addition to the object-oriented representation of the DGG, the toolkit implements utilities for extracting instrument footprints for simulation experiments. Our application requires aggregating hexagonal cells (BAUs) over regions commensurate with the ground footprint of a remote-sensing instrument (see the discussion of the OCO-2 and AIRS footprints in Sect. 3). For our prototype system, we have implemented two types of footprint extraction: nearest DGG cell and average of cells within a given radius. For each footprint location and radius, we use ASA to compute neighborhoods of DGG cells associated with footprints and to extract corresponding averages. In the case of footprints smaller than the DGG cell, we extract the value of the nearest-neighbor DGG cell. The resulting synthetic instrument observations are stored and made available for algorithm testing. Footprint plug-ins will allow us to specify satellite-footprint shapes, response curves, and measurement-error behavior to simulate how the actual instruments measure Earth and its atmosphere.

The DGGrid.py toolkit is designed to support the automated execution of simulation experiments. A single entry point allows the sets of parameters to be defined and systematically processed. Hence, testing and visualization can be carried out for different parameters, specified either for the conditional simulation or for the data-processing of observations.

#### Google Earth

Visualizing global data that have been computed on the sphere requires a globe upon which the rendering takes place. Although there are other “digital globe” displays, Google Earth offers a virtual-globe platform that is ubiquitous, accessible, and free. It also supports the visualization of global data as the user spins the globe and zooms in and out.

We use KML, which is the file format used to create Google Earth visualizations, to represent the hexagonal cells of a set of DGGs directly, as a list of coordinates that define the boundaries of the hexagonal cells. We then shade those cells’ interiors using a color palette to display the magnitudes of data associated with them. Representing each grid cell as a polygon in KML has the advantage of accurately displaying grid-cell boundaries at any scale, but it does not allow for the use of built-in multi-resolution pyramids for quick computation, display, and memory management. We deal with this by rendering small regions at finer resolutions (smaller polygons) and global data at coarser resolutions (larger polygons). The multi-resolutional nature of DGG allows us to easily group finer polygons and average them to create coarser polygons. We are also investigating how to render image pyramids for browsing and then how to transition to polygons when zooming in. Ideally, we would like to use pyramided arrays of hexagons, but this has been left for future research.

#### Integrating results into a GIS

Dealing with hexagonal-gridded data presents a problem of how to store data on hexagonal grids and how to move data in other formats into the hexagonally gridded environment. Typically, the original co-ordinate system on the surface of the sphere is based on latitude (from $-\,90^{\circ }$ to $+\,90^{\circ }$) and longitude (from $-\,180^{\circ }$ to $+\,180^{\circ }$). We have discussed in this chapter the computational procedure (Array Set Addressing, or ASA) we use for finding the cell, in the hexagonal DGG at any given resolution, that contains a given latitude–longitude location on the sphere. Most importantly, ASA allows the fast specification of neighboring cells, as well. The resulting ASA array in memory can be stored in a NetCDF container as two standard rectangular arrays, an even row image and an odd row image, along with the additional metadata needed to reconstruct the DGG in memory. Our hexagonally gridded output is geographic, but current GISs are not built to handle it efficiently. An open area of Geographic Information Science is to remedy this, and we believe that our article represents a beginning.

In the next section, we describe how we can use our approach to assess the performance of the Spatial Statistical Data Fusion (SSDF) algorithm. We shall eventually incorporate the ability to compute and display quantitative performance metrics from inside DDGrid.py, but, here, we focus on what can be learned by visually comparing the synthetic (i.e., simulated) input and the SSDF-algorithm output.

## Evaluating SSDF global estimates of CO$_{2}$

This section describes the specific implementation of our simulation–visualization system for evaluating the SSDF algorithm. SSDF produces optimal estimates of geophysical fields from two or more massive, heterogeneous, remote-sensing data sets. The methodology is similar to kriging and allows for input observations with different sampling characteristics and spatial supports. SSDF models, and subsequently leverages, spatial correlation in the data to produce optimal (minimum mean squared prediction error, unbiased) estimates of the underlying true fields; importantly, it also produces uncertainty measures (root-mean-squared prediction errors) of these estimates.

Here, we study the performance of the SSDF algorithm as it will be applied to data from two NASA instruments that measure carbon dioxide ($\hbox {CO}_{2}$) in the atmosphere: the Atmospheric Infrared Sounder (AIRS) and the Orbiting Carbon Observatory-2 (OCO-2). The AIRS instrument has been in orbit since mid-2002, and it observes mid-tropospheric $\hbox {CO}_{2}$ concentrations on circular footprints that are 90 km in diameter and are contiguous (Aumann et al. 2003). The OCO-2 instrument was launched in July 2014, and it observes total column $\hbox {CO}_{2}$ concentrations on contiguous trapezoidal footprints roughly 2 km in diameter (Eldering et al. 2012). Both instruments fly on satellites that are in polar orbit, observing the swaths of Earth along their respective tracks from pole to pole. The AIRS field of view across-track is about 1500 km, so its swaths are wide and the entire world is seen once every 3 days. The OCO-2 field of view across-track is only about 10 km, so its swaths are very narrow; the OCO-2 instrument never observes the whole world due to its narrow swath, but it repeats the same 233 globally distributed orbital paths every 16 days. Both instruments’ data are subject to high degrees of “missingness”, because neither can observe $\hbox {CO}_{2}$ in the presence of clouds.

To evaluate the performance of SSDF, we performed a simulation experiment using DDGrid.py. First, we generated a synthetic $\hbox {CO}_{2}$ field at fine spatial resolution using conditional-simulation technology (Sect. 2.1). The simulation was performed at DGG resolution-8 in which the BAU hexagons are 30 km in diameter.

The conditional simulation is calibrated to a coarser-simulated atmospheric $\hbox {CO}_{2}$ field, using the output of PCTM (Kawa et al. 2004) driven by analyzed meteorological fields from NASA’s Goddard Earth Observation System, version 4 (GEOS-4). In that model, the prescribed net surface fluxes of $\hbox {CO}_{2}$ were taken from the Carnegie Ames Stanford Approach (CASA; Randerson et al. 1997) model for biospheric fluxes, from Takahashi et al. (2002) for the monthly mean climatology for air-sea $\hbox {CO}_{2}$ exchange, from Erickson et al. (2008) for anthropogenic $\hbox {CO}_{2}$ emissions, and from the Global Fire Emission Database version 2 (GFED2; van der Werf et al. 2006) for wildfire and biomass-burning emissions. This model is herein referred to as PCTM for simplicity. The model has a horizontal resolution of 1$^\circ\, \times$ 1.25$^\circ$ with 25 vertical levels in the atmosphere. In the analysis presented here, we use the simulated fields from level 8 (approximately 5-km elevation, meant to represent the mid-troposphere) at 1800 GMT on April 15, 2006.

**Fig. 13:**
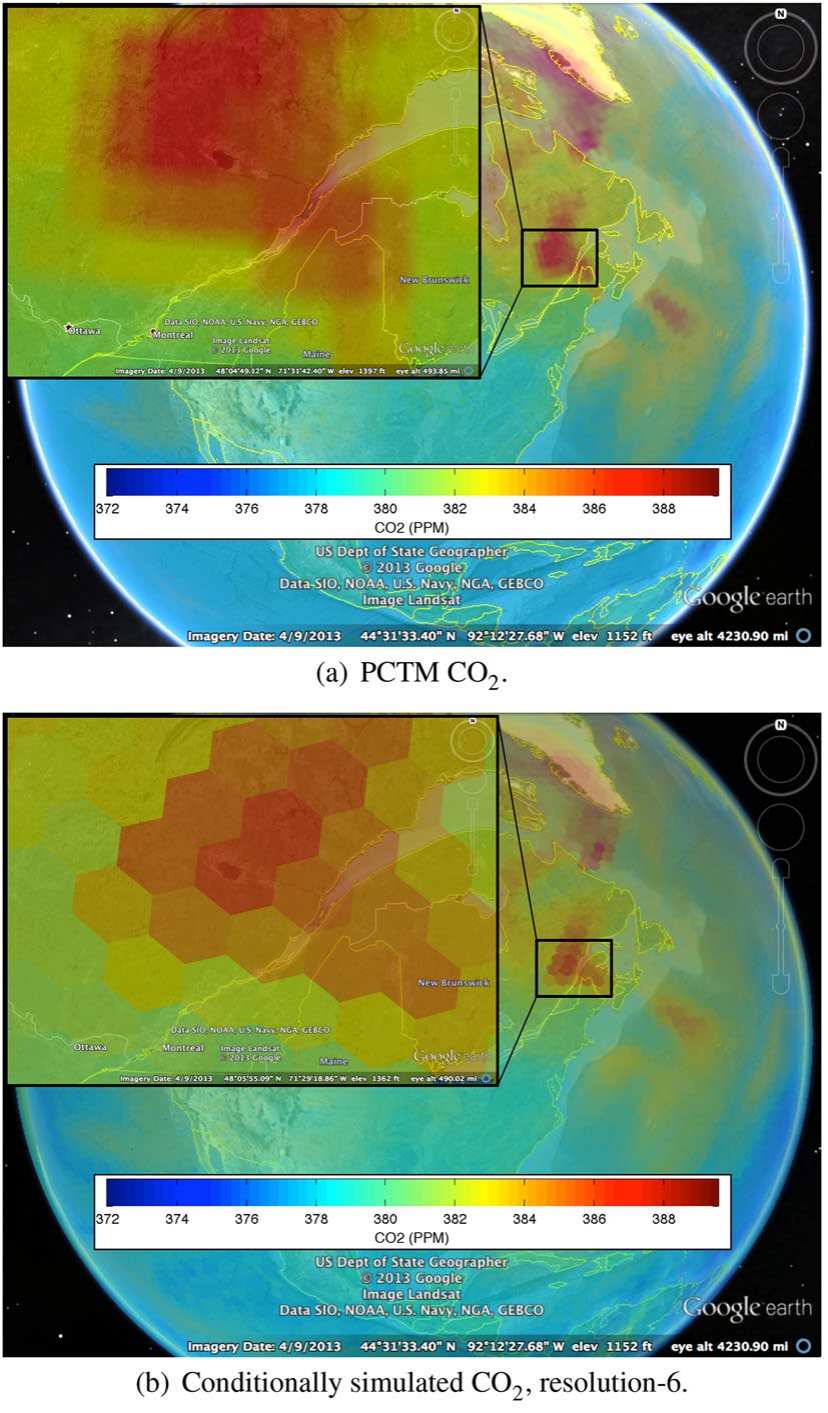
Coarse-resolution PCTM output and finer-resolution conditionally simulated $\hbox {CO}_{2}$ values (in ppm)

Figure 13a shows the coarse-resolution PCTM model output for the mid-troposphere; the region in the northeast of North America is featured. The PCTM resolution is approximately DGG-resolution-6 near the equator. This coarse resolution shows up as blockiness in Fig. 13a. Figure 13b is a global visualization of our conditional simulation at the finer resolutions. Although the data were produced at DGG resolution-8 (cells are 30 km in diameter), we have displayed the simulation output at the coarser DGG resolution-6 (cells are 120 km in diameter), to speed up display.

Here, we leveraged an important feature of our system. If we conditionally simulated at resolution-8 and aggregated to resolution-7 or to resolution-6, etc., we would obtain a process whose statistical properties would be the same as those from direct conditional simulation at the respective resolutions. The visualizations in Fig. 13a, b show nearly identical features, as they should have given the constraint that the conditionally simulated field at all resolutions must aggregate to reproduce the values on the PCTM grid.

In the second step, we sampled the conditionally simulated field to create *synthetic observations* analogous to what AIRS and OCO-2 would “see.” We started with the centers of actual AIRS and OCO-2 footprints. For AIRS, we used the locations of non-missing footprints for a representative 3-day period. To create synthetic AIRS observations, we averaged simulated values for all 30-km hexagons (DGG resolution-8) with centers falling within a 45-km radius of the actual center of the AIRS footprint. In the case of OCO-2, whose footprint is smaller than the resolution-8 hexagon, we took the value of the simulated data for the hexagon with center nearest to the center of the OCO-2 footprint. We used three representative days of simulated orbit tracks provided to us by the OCO-2 team at NASA’s Jet Propulsion Laboratory.

**Fig. 14:**
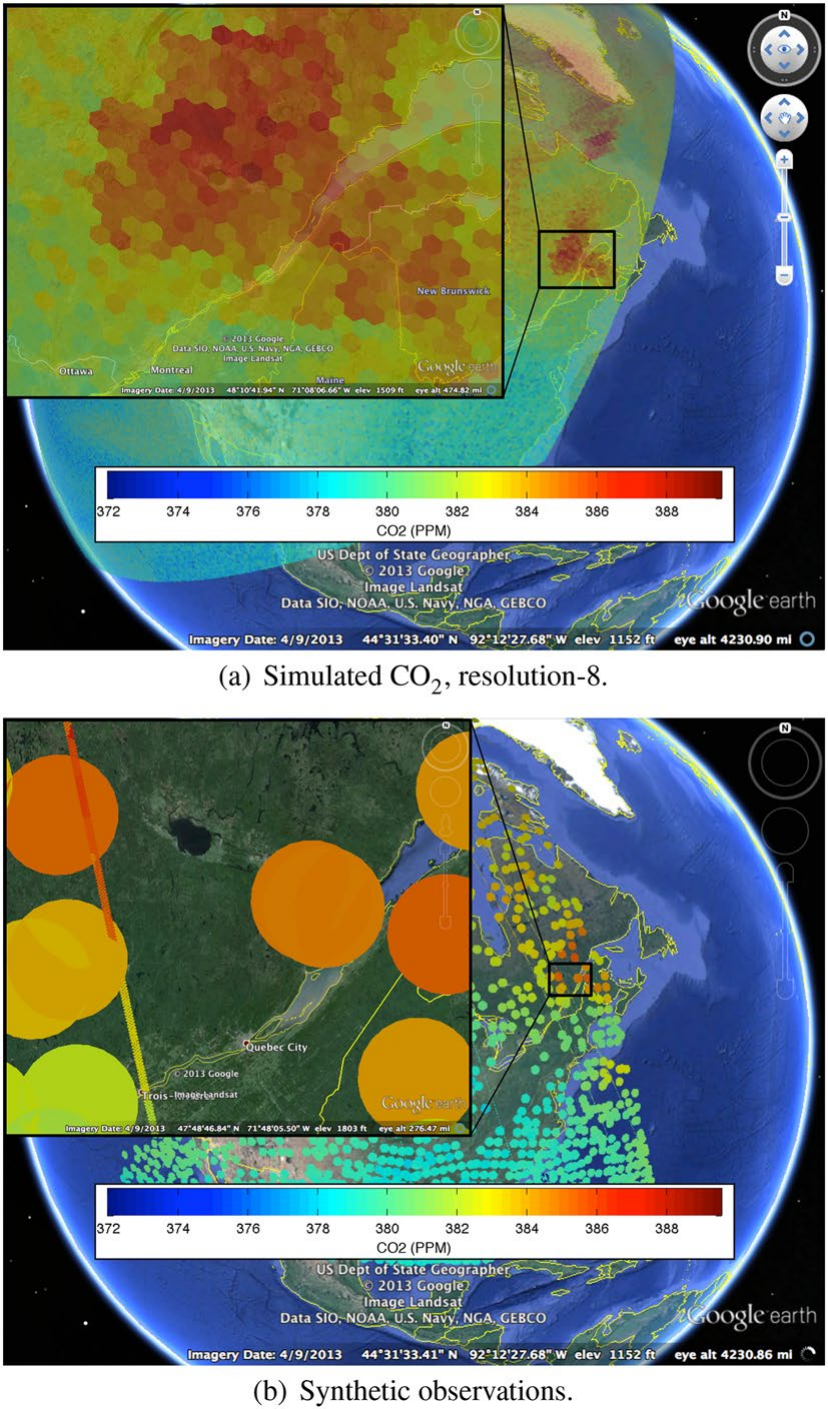
Conditionally simulated $\hbox {CO}_{2}$ and the synthetic AIRS and OCO-2 observations during a 3-day period

Figure 14a shows the simulated field at DGG resolution-8 for a wedge of Earth, with an inset that zooms in on eastern New England and Quebec, in order that the 30-km hexagons are clearly visible. Synthetic observations for AIRS and OCO-2 are shown in Fig. 14b. The main image shows the locations and values of AIRS observations for a subset of Earth’s surface, color-coded according to their simulated values. From the inset, we get a better view of eastern New England and Quebec—the circles show the locations and sizes of the AIRS observations. The thin, almost vertical, strip represents the OCO-2 orbit track, although there is a representation issue here, because the strip is made up of 2-km-diameter regions with values taken from the nearest 30-km hexagon. The size mismatch between AIRS and OCO-2 footprints would render the OCO-2 footprints invisible if we did not use the zoom in Fig. 14b. The OCO-2 footprints are also color-coded according to their simulated values.

**Fig. 15:**
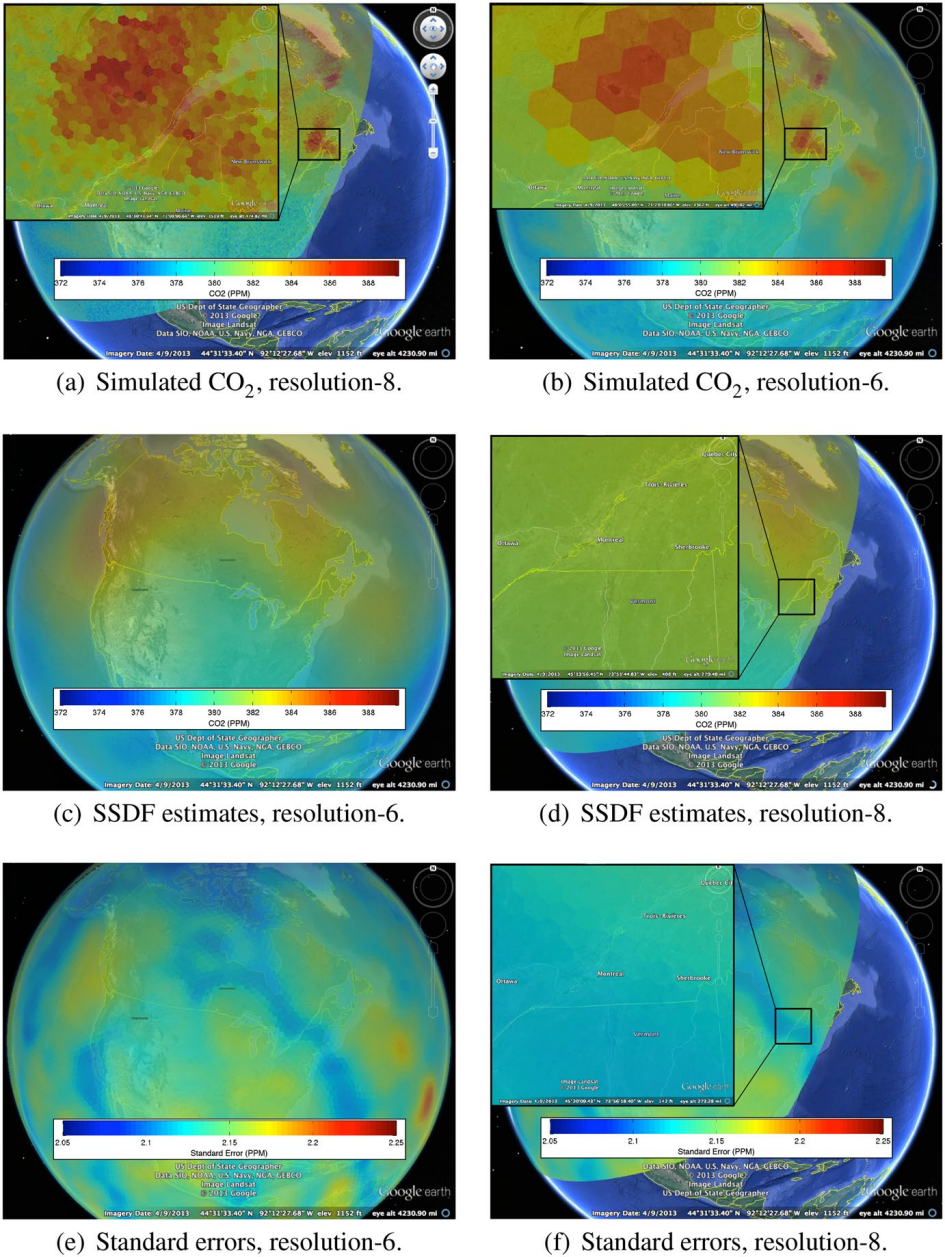
Spatial–Statistical Data Fusion (SSDF) from (synthetic) observations obtained from the AIRS and OCO-2 instruments

Finally, we apply SSDF to estimate a BAU-contiguous field of $\hbox {CO}_{2}$ concentrations obtained from both synthetic AIRS and synthetic OCO-2 observations, where SSDF uses optimal spatial weights (Nguyen et al. 2012). Our estimates are produced on BAUs at 30-km spatial resolution (DGG resolution-8). Figure 15d, f shows the fused estimates and corresponding standard errors at resolution-8 for the same wedge of Earth as in Fig. 14a, and with high-resolution insets. Figure 15c, e shows the corresponding global views produced by aggregating the resolution-8 SSDF results up to resolution-6. Figure 15a, b is duplicates of Figs. 13b and 14a for easy comparison.

Exploratory evaluation of SSDF might include visually comparing Fig. 15d to a and comparing Fig. 15c to b. The former is a regional comparison at a finer resolution, and the latter is a global comparison at a coarser resolution. Both comparisons should be considered in light of the standard-error maps that correspond to the spatial-statistically fused estimates. These are shown in Fig. 15f and e, respectively.

One can make a number of observations about SSDF based on these visualizations. At the global scale, the SSDF estimates of $\hbox {CO}_{2}$ in Fig. 15c give a smoother impression than the simulated $\hbox {CO}_{2}$ process given in Fig. 15b. The standard errors in Fig. 15e show features that do not appear to correspond to features in the estimates themselves, but they do have some similarities to the simulated output in Fig. 15b. Recall that the input to SSDF is made up of sparse synthetic footprints like those shown in Fig. 14b. This accounts for the smoothing in the fused estimates, and it also influences geographic patterns in the standard errors. At the finer spatial scale (Fig. 15d), the smoothing is even more pronounced, and it is accompanied by similar smoothing in the standard-error map (Fig. 15f). This is in sharp contrast to the spatial heterogeneity of the resolution-8 simulated field in Fig. 15a and is due to the sparsity of the synthetic observations in the region of the inset. Our simulation–visualization experiments illustrate that SSDF estimates are likely to be more useful on global scales than on regional ones if the instrument data are geographically sparse. This is not surprising, and it could have been anticipated with some knowledge of how SSDF works (i.e., it is akin to kriging), but this visualization tool makes it possible to understand how problematic this is for specific regions of interest.

## Conclusion

We have built an initial version of a simulation, analysis, and visualization system, along the lines of a GIS, which ties the computation and visualization environment to the representation of the underlying data in nested, discrete global grids. In our implementation, the underlying fine-resolution data are produced using a spatial–statistical conditional-simulation methodology. The methodology constrains the simulation output to reproduce features of a physical model that was constructed from scientific knowledge about the structure of the true physical process.

We have developed a python toolkit to implement instrument-like sampling of the simulated field, manage interfaces between component technologies, and augment them where necessary. We have demonstrated how our system can be used to visualize and better understand the behavior of a global data processing algorithm, SSDF, over different spatial scales. This is possible, because the simulated field obeys hierarchical aggregation consistency, so that coarse-resolution fields can be derived in a statistically controlled way from fine-resolution fields. This should be mirrored by the upscale-pyramiding capability within our visualization platform. Further research would enable a downsample-pyramiding capability that would generate fine-resolution simulated fields for limited regions and display them in near-real time. This infrastructure was implemented for the SSDF algorithm, but other computational algorithms whose performance depends on fine-resolution spatial structure can also be evaluated.
